# Tobacco Smoking and Mindful Self-Care among Workers

**DOI:** 10.1192/j.eurpsy.2026.10816

**Published:** 2026-07-31

**Authors:** I. Sellami, A. Abbes, A. Feki, A. Hrairi, M. A. Ghrab, M. L. Masmoudi, M. Hajjaji, K. Jmal Hammami

**Affiliations:** 1 occupational medicine; 2 Rheumatoloy, Hedi Chaker Hospital, sfax, Tunisia

## Abstract

**Introduction:**

Tobacco smoking remains a major public health concern, particularly among workers exposed to occupational stress. In addition to its well-established physical risks, smoking may impair mindful self-care practices, diminishing individuals’ capacity to manage stress and maintain healthy behaviours.

**Objectives:**

Our study aimed to examine the association between tobacco smoking and mindful self-care among workers.

**Methods:**

We conducted a descriptive, analytical, cross-sectional survey among workers. Data on sociodemographic and professional characteristics, smoking behaviours, and self-care practices were collected, with mindful self-care assessed using the Mindful Self-Care Scale (MSCS).

**Results:**

Our population comprised 497 workers with a mean age of 42.2 ± 11.1 years. The mean of tenure of job was 14.5 ± 10.6 years. We found that 44.1% of participants were active smokers. The mean of mindful self-care score was 99.2 ± 34.6. The mean of mindful relaxation, physical care, self-compassion and purpose, supportive relationships, supportive structure and mindful awareness were respectively 11.1 ± 5.7, 18.4 ± 7.5, 17.2 ±8.9, 14.3±7.6, 11.8±6.7 and 11.3±6.3. Tobacco smoking was associated to low score of supportive relationships (p= 0.03).

**Conclusions:**

This study highlights that tobacco smoking is associated with weaker supportive workplace relationships, which in turn is linked to lower levels of mindful self-care. These associations may contribute to reduced worker productivity, underscoring the importance of workplace smoking prevention and health promotion initiatives.

**Disclosure of Interest:**

None Declared

